# One-Step Synthesis of Poly(L-Lactic Acid)-Based Soft Films with Gas Permselectivity for White Mushrooms (*Agaricus bisporus*) Preservation

**DOI:** 10.3390/foods12030586

**Published:** 2023-01-30

**Authors:** Tao Sun, Junxia Bian, Yangyang Wang, Jian Hu, Xueyan Yun, Eerdunbayaer Chen, Tungalag Dong

**Affiliations:** 1 College of Food Science and Engineering, Inner Mongolia Agricultural University, 306 Zhaowuda Road, Hohhot 010010, China; 2 Hohhot Huimin District Center for Disease Control and Prevention, Hohhot 010030, China

**Keywords:** *Agaricus bisporus*, one-step synthesis, poly (L-lactic acid), soft packaging film, O_2_/CO_2_ atmosphere, food shelf life

## Abstract

Proper packaging can extend the shelf life and maintain the quality of mushrooms during storage. The purpose of this study is to investigate the preservation of *Agaricus bisporus* using copolymer-modified poly (L-lactide-co-butylene fumarate) and poly (L-lactide-co-glycolic acid) (PLBF and PLGA) packaging. Shelf life and quality were evaluated over 15 days of storage of *Agaricus bisporus* at 4 ± 1 °C and 90% relative humidity, including weight loss, browning index (BI), total phenolics (TP), ascorbic acid (AA), malondialdehyde content (MDA), electrolyte leakage rate (EC), and superoxide dismutase (SOD) and catalase (CAT). The results showed that mushrooms packaged in PLBF films exhibited better retention in BI, TP, and AA than those with PLLA, PLGA, or polyethylene (PE) films. They can reduce the rate of weight loss, EC, and MDA, which in turn increases the activity of SOD and CAT. PLBF and PLGA have substantially improved flexibility in comparison with PLLA. They also significantly reduced oxygen (O_2_) and carbon dioxide (CO_2_) permeability and changed the gas permeability ratio. These positive effects resulted in the effective restriction of O_2_ and CO_2_ in these packages, extending the post-harvest storage period of white mushrooms.

## 1. Introduction

*Agaricus bisporus* (button mushroom) is popular in the global food market and accounts for 30% of the world’s total mushroom production [1]. Their excellent texture is characterized by their high nutritional value, characteristic flavors, and high protein and fat contents compared to other vegetables [2]. However, the potential market for this type of mushroom is somewhat inhibited by its short shelf life [3], which is 1~3 days at room temperature and 5∼8 days under refrigerated conditions [4]. Without the cuticle, high metabolic activity, high respiration rates, and dehydration are responsible for the rapid decay of the mushroom [5]. As it is separated from the water and nutrients (organic matter, minerals) in the culture medium, harvested *Agaricus bisporus* is subject to the influence of the external environment, which leads to a constant depletion of nutrients and deterioration in storage quality [6]. Upon leaving the culture medium, a mushroom begins to respire, a process in which tissues decompose faster to maintain their own physiological metabolism, including breaking down cellular walls and producing reactive oxygen species [7]. Mushroom cell walls are composed of chitin and β-glucan, and as a result of enzyme action, they gradually decompose; firmness is rapidly destroyed, and tissue degeneration occurs more rapidly due to autolysis [8]. Reactive oxygen species and free radicals produced by respiration accelerate the oxidation of phospholipids on cell membranes, which leads to the production of peroxide malondialdehyde (MDA) and electrolyte leakage [9,10]. If the O_2_ content in the environment around the bivalve mushroom is reduced to less than 1% (O_2_ < 1%, CO_2_ > 5%), the respiration rate is inhibited, which can prevent the accumulation of MDA while reducing oxidative damage to the mushroom [11]. Therefore, to extend the shelf life of *Agaricus bisporus*, it is initially necessary to ensure that it remains in a normal state of activity, and on this basis, to create an environment with extremely low oxygen content inside the package to inhibit its respiration, creating a slower metabolic environment and thus retaining freshness.

Poly (L-lactic acid) (PLLA) is a fully biodegradable polymer derived from sustainable sources. PLLA is becoming increasingly popular as an alternative to synthetic plastics in the packaging sector due to its desirable characteristics of high transparency, high stiffness, high strength, and thermal stability [12,13,14]. Nevertheless, PLLA’s poor gas barrier properties present a challenge and a need for further research in order for PLLA to compete with fossil-based plastics in such essential functions [15]. Many techniques can be used to enhance the gas barrier properties of PLLA. In Ali’s study, PLLA was used to prepare PLA-based polyurethane (PLAPU) films containing PCL diol as a softening agent. An acquired blend consisting of PLAPU and PCL at a ratio of 1:3 demonstrated the best mechanical and gas barrier properties [16]. Genovese’s group synthesized novel PLLA-based chain-extended triblock copolymers, and the results showed that copolymerization led to better mechanical and barrier properties in comparison with PLLA [12]. Thus, by altering the polymerization structure of PLLA, we can enhance its mass transfer properties, which improves its adsorption properties as well as its O_2_ and/or CO_2_ barrier properties [17,18]. This will help maintain food quality and safety since the rate of typical food degradation reactions (such as oxidation, spoilage, and deterioration) will be significantly reduced.

Equilibrium modified atmosphere packaging (EMAP) reduces the product’s respiration rate, preserving its quality and extending its shelf life [19]. EMAP has become increasingly common in recent years, where products are packaged using selected types of film, and the required atmosphere is formed naturally due to product respiration and diffusion of gases through the film [20,21]. EMAP is richer in CO_2_ and lower in O_2_ than air, with the potential to reduce respiration rate, spoilage, and physiological changes [22]. EMAP is more adaptable than conventional packaging and is, therefore, a better solution for plants undergoing respiration; as a consequence, researchers have gradually turned their attention to the development of EMAP over the past few years [23]. The study of the effect of EMAP on the quality changes in mushrooms has found that low O_2_ levels and/or high CO_2_ levels produced by the packaging material have been effective in improving the storage quality of *Agaricus bisporus*, reducing browning production, enhancing antioxidant enzyme activity, and preventing ageing [11,24,25]. For this effect to be achieved, it will be necessary to improve the O_2_ barrier properties and O_2_ to CO_2_ transmission ratio of the materials used for EMAP. Commercial modified atmosphere packaging used in the cryopreservation of *Agaricus bisporus* has been made from nondegradable plastics, which can cause environmental pollution. Researchers are now working on the preparation of EMAPs using degradable plastics [22,26,27,28].

Accordingly, the development of modified PLLA to prepare EMAP is an opportunity to increase the shelf life of mushrooms. To our knowledge, there has been little research conducted on the application and comparison of modified PLLA films to enhance the shelf life and quality of *Agaricus bisporus*. Due to its low gas barrier and poor flexibility, PLLA is difficult to use directly in food packaging, especially for irregularly shaped foods such as mushrooms. When PLLA is used for mushroom packaging, it usually breaks due to its brittleness, and its barrier properties do not meet the needs of packaging mushrooms. The novel approach we propose is to introduce poly(butylene fumarate)(PBF) or poly(glycolic acid)(PGA) blocks with high barrier properties into PLLA. PBF, which is a biobased unsaturated aliphatic polyester with properties similar to those of the traditional biobased saturated polyester poly (butylene succinate) (PBS), is attracting increasing interest because it has excellent processing characteristics and low production costs. The excellent flexibility of PBF may bring better mechanical properties to PLLA, while its similar structure to PSB also indicates that it may promote the barrier property of PLLA. [29,30,31,32]. While the excellent barrier and processing properties of PGA and its composites have been demonstrated to make them suitable for food packaging, their promotion for use in this application has been difficult due to high production costs. [33,34].

In this study, we developed PLLA-based films by a one-step synthesis method that included the addition of small amounts of oligo (butylene fumarate) (OBF) or glycolic acid (GA). Our work investigated the effects of OBF or GA on the properties of PLLA films, including flexibility properties as well as gas-selective permeability of the films. Additionally, their application in the preservation of white mushrooms under refrigeration at 4 °C was studied to determine the differences in commercial value during storage of white mushrooms under different atmospheric conditions created by PLBF and PLGA and the effect of films with different barriers and selective gas permeability on the quality of white mushrooms.

## 2. Materials and Methods

### 2.1. Materials

*Agaricus bisporus* was harvested from the Shengle plantation in Hohhot City Hellinger County. PE film, purchased from the Hohhot Hualian supermarket, was approximately 30 ± 0.5 μm thick. The CDP and OP of the PE film were 5.94 × 10^−12^ cm^3^/m·s·Pa and 1.97 × 10^−12^ cm^3^/m·s·Pa, respectively.

L-lactic acid (L-LA; 90 wt % in water; Aladdin), glycolic acid (GA; 98%; Aladdin Bio-Chem Technology Co., Ltd. Shanghai, China), butane-1,4-diol (BDO; 99%; Aladdin), fumaric acid (FA; 99%; Sigma-Aldrich (SHANGHAI) Trading Co., Ltd. Shanghai, China), tin(II) chloride dihydrate (SnCl_2_·2H_2_O; 99.99%; Aladdin), hydroquinone (99%; Sigma-Aldrich), and P-toluenesulfonic acid (TSA; 99%; Aladdin) were used. Unless otherwise specified, all the other reagents used in this study were obtained from Sinopharm Chemical Reagent Co., Ltd. (Shanghai, China) and were analytical grade.

### 2.2. Film Preparation

#### 2.2.1. Synthesis of Oligo (L-Lactic Acid) and Poly (L-Lactic Acid)

Lactic acid (700 g) was added into a 2 L reaction flask equipped with a mechanical stirrer and nitrogen inlet. The flask was sequentially degassed by a vacuum pump and purged with high purity Ar 3 times to ensure an Ar atmosphere during the reaction. The system was first heated to 110 °C for approximately 1 h under 400 mbr, then to 150 °C for approximately 2 h under 130 mbr, and then degassed to 40 mbr for 4 h; reduced pressure ensured the removal of water monomers. The products were finally poured out of the flask, resulting in the production of oligo (L-lactic acid) (OLLA).

The synthetic catalyst (SnCl_2_·2H_2_O/TSA, 0.5 wt% of gross weight) was added to the OLLA, warmed to 180 °C, and reacted under vacuum for 12 h. After the reaction was stopped, the product was removed from the reactor and cooled to room temperature naturally. The product is the PLLA sample.

#### 2.2.2. Synthesis of Oligo (Butylene Fumarate)

A total of 64.4 g of fumaric acid, 50 g of 1,4-butanediol, and a proper amount of hydroquinone (0.5 wt%) were added into a 500 mL reaction flask equipped with a mechanical stirrer and nitrogen inlet. The flask was sequentially degassed by a vacuum pump and purged with high purity Ar 3 times to ensure an Ar atmosphere during the reaction. Then, the system was heated to 150 °C for approximately 2 h under continuous Ar purge and was subsequently degassed to a pressure of 10 mbr for 4 h. The products were poured out of the flask, and the oligo (butylene fumarate) (OBF) was obtained.

#### 2.2.3. Synthesis of the PLGA Copolymer

PLGA synthesis was conducted as follows. Appropriate masses of OLLA (92 wt%), GA (8 wt%), and catalyst (SnCl_2_·2H_2_O/TSA, 0.5 wt% of gross weight) were weighed into a 2-L batch reactor equipped with a mechanical mixer, a nitrogen inlet, temperature control by oil circulation, and a vacuum pump. The reactor’s temperature was raised gradually from ambient temperature to 180 °C within 10 min; subsequently, the pressure was reduced stepwise to 30 mbar within 5 min. After a total reaction time of 20 h, the polymer was removed from the reactor and given the designation PLGA.

#### 2.2.4. Synthesis of the PLBF Copolymer

PLBF synthesis was conducted as follows. Appropriate masses of OLLA (92 wt%), OBF (8 wt%), and catalyst (SnCl_2_·2H_2_O/TSA, 0.5 wt% of gross weight) were weighed into a 2 L batch reactor equipped with a mechanical mixer, a nitrogen inlet, temperature control by oil circulation, and a vacuum pump. The reactor’s temperature was raised gradually from ambient temperature to 180 °C within 10 min; subsequently, the pressure was reduced stepwise to 30 mbar within 5 min. After a total reaction time of 20 h, the polymer was removed from the reactor and given the designation PLBF.

#### 2.2.5. Preparation of Films

PLLA, PLGA, and PLBF samples (0.9 g) were dissolved in chloroform (50 mL) used for film mold, and the films were removed from the mold after the chloroform evaporated and allowed to dry for 30 days.

### 2.3. Measurements and Characterization

#### 2.3.1. Attenuated Total Refection Fourier Transform Infrared Spectroscopy

An IRAffinity-1 spectrophotometer (SHIMADZU (CHINA) Co., Ltd. Shanghai, China) equipped with a diffuse reflectance accessory was used to measure ATR-FTIR spectra with a resolution of 4 cm^−1^ and 64 scans from 700–4000 cm^−1^.

#### 2.3.2. Nuclear Magnetic Resonance

The ^1^H NMR spectra of the polymers were recorded on a 400 MHz Bruker Advance 2B spectrometer in a solvent of deuterated chloroform (CDCl_3_). The copolymer composition was evaluated with ^1^H NMR. ^1^H NMR signals of PLGA: 1.57 (–CH_3_), 4.85 and 4.60 (–CH_2_–), 5.17 (–COCH(CH_3_)O–). ^1^H NMR signals of PLBF: 1.57 (–CH_3_), 1.75 (–CH_2_–), 4.22(–CH_2_–), 5.17 (–COCH(CH_3_)O–), 6.88(–CH=CH–). 

#### 2.3.3. Gel Permeation Chromatography

Molecular weights were determined using gel permeation chromatography (GPC, Waters Co., Milford, MA, USA). A 1.0 mL/min flow rate of THF was used as the eluent; the column temperature was 30 °C. The polydispersity was also determined using gel permeation chromatography.

#### 2.3.4. Tensile Test

The tensile strength (σ_t_, MPa), Young’s modulus (*E*, MPa), and elongation at break (ε_b_, %) were measured on a tensile testing machine (XLW(EC), Labthink, Jinan, China). According to ASTMD882-09, the film samples were cut into rectangular strips (28 mm × 5 mm) and tested on the machine at a speed of 10 mm/min. Seven parallel samples were taken from each group, and the average test results were taken.

#### 2.3.5. Gas Barrier Properties

##### Oxygen and Carbon Dioxide Permeability

The O_2_ and CO_2_ permeability of the films (OP and CDP) was determined by a gas permeability tester (VAC-V2, Labthink, Jinan, China) in accordance with GB/T1038-2000. The test was performed under environmental conditions (temperature: 5 °C, RH: 50%). The transmission rates (TRs) of CO_2_ and O_2_ were obtained directly from the test results, and the permeability of CO_2_ and O_2_ (cm^3^/m^2^·d) was calculated using the following formula.

(1) $$\mathrm{OP}/\mathrm{CDP}=\frac{\mathrm{OTR}/\mathrm{CDTR}\times D}{\Delta P}$$

*D* represents the thickness of the films, and Δ*P* represents the gas pressure difference between the two sides of the film (Pa), which was a standard atmospheric pressure (1.01 × 10^5^ Pa).

### 2.4. Application for Agaricus bisporus Packaging

*Agaricus bisporus* mushrooms were precooled after being picked under 2 ± 1 °C for two hours before testing and then those mushrooms that were round, of good hardness, white in color, unopened, free of pests, and without mechanical damage were selected for testing. The mushrooms were packaged in PE, PLLA, PLBF, and PLGA bags, with each bag containing 80 ± 5 g of mushrooms. The packaged mushrooms were stored at 4 °C for 15 days, and their quality was tested every three days to evaluate whether they were protected. At the same time, 80 ± 5 g of mushrooms were weighed in each of 6~8 clean trays as a CK group and stored in the same environment as the rest of the packaged group to achieve a similar storage effect. A randomly selected group was tested against the packaged group on each test day.

#### 2.4.1. Headspace Analysis

The gas composition of the bags was measured using a headspace gas analyzer (Type 6600, Systech, Oxford, UK). The instrument was calibrated before the test, and each group was measured 3 times in parallel. The CO_2_ and O_2_ concentrations in % were displayed directly on the instrument’s screen. 

#### 2.4.2. Weight Loss

The weight loss rate of the samples was calculated by following formula.

(2) $$Weight\;Loss=\frac{m_{1}-m_{2}}{m_{1}}\times 100\%$$

where *m*_1_ (g) represents the initial weight of the mushrooms, and *m*_2_ (g) represents the weight on the day of the experimental test. Three bags of mushrooms from each group were measured at a time.

#### 2.4.3. Browning Index

The color of mushrooms during storage was measured using a portable colorimeter (CR-20, Minolta Co., Ltd., OSAKA, Japan). Three mushrooms were randomly selected from each packaged group after calibration using the accompanying standard white plate. As a result of testing the center of the mushroom cap, *L* (lightness), *a* (redness), and *b* (yellowness) values were gathered. The resulting browning index (BI) can be calculated as follows [10].

(3) $$\mathrm{BI}=\frac{100\times \left(X-0.31\right)}{0.172}$$

where

(4) $$X=\frac{a^{*}+1.75L^{*}}{5.645L^{*}+a^{*}-3.012b^{*}}$$

#### 2.4.4. Total Phenolics and Ascorbic Acid

The total phenolics (TP) of the samples were determined by the method developed by Pirie and Mullins in 1976, where gallic acid was used as a standard solution [35]. The ascorbic acid (AA) content was determined using a 2,6-dichlorophenol indophenol (DCPIP) titration method [36].

#### 2.4.5. Malondialdehyde Content and Electrolyte Leakage Rate

The mushroom’s internal tissue (1 g) was ground and homogenized with 10 mL of 100 g L^−1^ trichloroacetic acid (TCA) solution and centrifuged at 10,000× *g* and 4 °C for 20 min to obtain the mushroom extract. The extract was mixed with 4 mL of 0.67 g/100 g thiobarbituric acid (TBA), incubated in boiling water for 20 min, cooled, and centrifuged again to collect the supernatant. The extraction was replaced by 4 mL of 0.67 g/100 g TBA and 4 mL of 100 g L^−1^ TCA solution as the control blank. The absorbance of the supernatant was measured at 532 nm (*OD*_532_), 600 nm (*OD*_600_), and 450 nm (*OD*_450_). The MDA content could be calculated as follows:

(5) $$\mathrm{MDA}\left(\mathrm{nmol}\;g^{-1}\;\mathrm{FW}\right)=\frac{\left[6.45\times \left(OD532-OD600\right)-0.56\times OD450\right]\times Volume}{Sample\;mass}$$

The electrolyte leakage rate was analyzed following a previously described method [37]. Approximately 2 g of mushrooms was cut into small cubes, washed 3 times with deionized water, and dried. The plates were immersed in 25 mL of deionized water at 25 °C for 30 min. The electrical conductivity (γ0) of the suspension solution was determined immediately using an electrical conductivity meter (DDSJ-308A, Leici Instrument Co., Shanghai, China). The electrical conductivity (γ1) was measured after the electrolyte was boiled for 10 min and cooled to 25 °C. The electrolyte leakage rate was calculated as follows.

(6) $$\gamma \left(\%\right)=\frac{\gamma 0}{\gamma 1}\times 100\%$$

#### 2.4.6. Enzyme Activity

Catalase (CAT) activity was measured following Cao and Jiang’s method with some modifications [38]. CAT was measured in a reaction mixture of 1.5 mL of 50 mmol/L potassium phosphate buffer (pH 7.0), 0.3 mL of 50 mmol/L H_2_O_2_, and 0.2 mL of enzyme extract. The absorbance at 240 nm was recorded once every 30 s for 3 min. One unit of enzymatic activity was defined as the amount of the enzyme that caused a change of 0.01 in absorbance per minute and caused changes in the substrate. The specific CAT activity was expressed as U/g on a protein basis.

Superoxide dismutase (SOD) activity was measured according to the method of Cao [38]. The change in absorbance at 560 nm was recorded. SOD activity was expressed as U/g fresh weight (FW), where one unit of SOD activity was defined as the amount of enzyme that caused 50% inhibition of nitroblue tetrazolium (NBT). 

### 2.5. Statistical Analysis

All quantitative data were statistically analyzed and presented as the means ± standard deviations (SD) of three independent treatments. One-way analysis of variance (ANOVA) was performed using IBM SPSS Statistics 22 to compare the means by Duncan’s test. Differences among groups were considered significant at *p* < 0.05.

## 3. Results and Discussion

### 3.1. Characterizations of Films

#### 3.1.1. Structural and Molecular Characterization of Films

**Figure 1:**
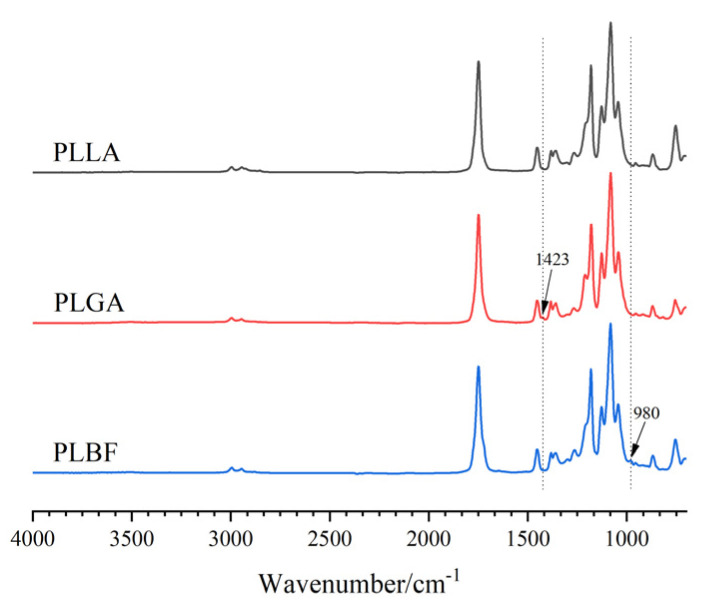
ATR-FTIR spectra of PLLA, PLGA, and PLBF.

Figure 1 presents the ATR-FTIR spectra of PLLA, PLGA, and PLBF. All three polymers in the spectrum exhibit the carbonyl group (C=O) stretching mode band as part of the PLLA unit at 1750 cm^−1^ [39]. This peak is the amorphous absorption peak of the PLLA ester carbonyl group, indicating that the PLLA unit in all three materials exhibits an amorphous state at this time. It was also observed at 1180 cm^−1^ and 1080 cm^−1^ that there was a robust asymmetric stretch vibration peak of the ether bond (C-O-C) and a symmetric stretch vibration absorption peak. Similarly, the characteristic peak at 1423 cm^−1^ in the PLGA spectra was assigned to the (-CH_2_-) bending in GA units [40], while the characteristic peak at 980 cm^−1^ in the PLBF spectra was assigned to the C-H bending on trans CH=CH in PBF units [41]. This result showed that PLGA and PLBF copolymers were successfully synthesized.

**Figure 2:**
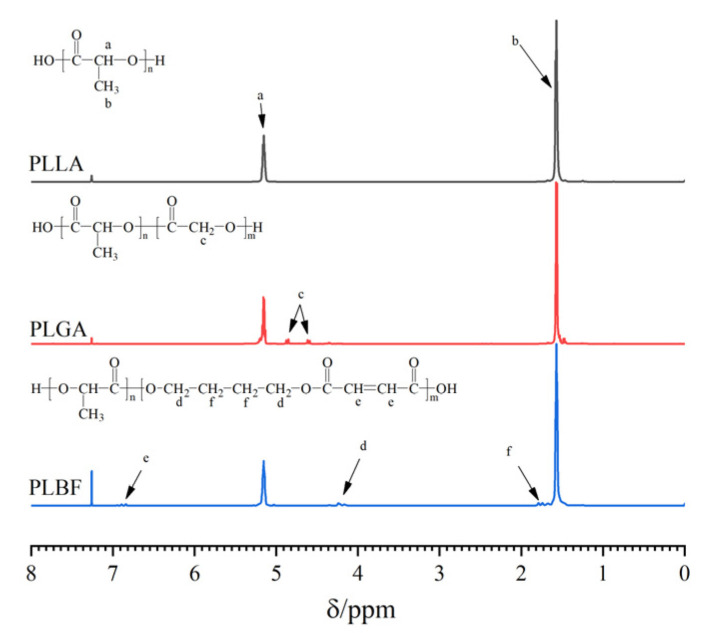
^1^H NMR spectra of PLLA, PLGA, and PLBF.

The chemical shift values measured in this study are analogous to those reported in the literature for PLGA [42] and PBF [41] copolymers. As seen in Figure 2, the (2H, 4.85 and 4.60 ppm) peaks of GA units are multiple peaks, separated by a wide gap, indicating that in the one-step melt copolymerization of GA and LA, large molecules of random polymers are the main components of polymers, and the different sequences complicate peak splitting [43]. However, in the one-step manufacture of the PLBF copolymer, this phenomenon also exists, indicating that PLBF is also a random polymer. There are several explanations for such complex peak splitting, including the lower weight of GA and BF units and the complex sequences in the molecules in the PLGA and PLBF molecular chains.

We evaluated the molecular weight, polydispersity (Pd), and relative mass ratio of polymers by GPC and ^1^H NMR, as shown in Table 1. There is a range of PLLA units between 92.5 and 91 wt%, and all copolymers possess molecular weights greater than 4.0 × 10^4^, indicating acceptable film formation characteristics. There was a slight difference in the percentage of each feedstock unit determined by NMR compared to the feedstock additions during the synthesis, which may be attributed to the PLLA homopolymer present in the systems during the synthesis. However, it is worth mentioning that all copolymers have relatively low Pd values, suggesting a narrow distribution of molecular weights. The above results demonstrated that PLLA, PLGA, and PLBF copolymers with high molecular weights were successfully synthesized in this study.

#### 3.1.2. Mechanical Properties

The mechanical properties of packaging materials are essential performance parameters and critical performance indicators in *Agaricus bisporus* packaging. Table 2 contains the parameters associated with tensile strength (σ_t_), elongation at break (ε_b_), and Young’s modulus (*E*) for PLLA, PLGA, and PLBF films. PLLA films are brittle materials with a tensile strength of 40.3 MPa and an ε_b_ value of 5.7%; however, the ε_b_ values of the PLGA and PLBF films increased to 121.1% and 139%, respectively, while their σ_t_ values decreased to 23.3 and 25.6 MPa, respectively. Meanwhile, the *E* value of PLLA films was 1352 MPa, while the *E* values of PLGA and PLBF copolymer films decreased to 525 and 559 MPa, respectively, which was 61.2% and 58.7% less than that of PLLA films, showing a tomographic decrease.

Due to the addition of PGA and PBF units, the PLBF and PLGA copolymers have a higher degree of flexibility than PLLA, which is probably caused by a disruption of the regularity of the PLLA molecular chain. This decreases the σ_t_ values of PLGA and PLBF, consequently increasing flexibility [44]. Furthermore, the PLGA and PLBF films are in an amorphous state after copolymerization, thereby reducing the intermolecular forces, resulting in increased movement of molecular chain segments within the material, resulting in a significant decrease in *E* values.

#### 3.1.3. Gas Permeability Properties

Gas permeability is a crucial factor influencing the freshness retention of equilibrium modified atmosphere packaging. Table 3 reveals OP, CDP, and P_C/O_ for the copolymer films. It can be seen from Table 3 that CDP, OP, and P_C/O_ for PLLA were 4.51 × 10^−12^ cm^3^/m·s·Pa, 1.50 × 10^−12^ cm^3^/m·s·Pa, and 3.0. In contrast, when GA and PBF were introduced into PLLA, CDP and OP decreased outstandingly, and P_C/O_ increased significantly. It is worth noting that PLBF had the lowest CDP value of 2.08 × 10^−12^ cm^3^/m·s·Pa and the lowest OP value of 0.46 × 10^−12^ cm^3^/m·s·Pa; only 46% and 30% of PLLA, respectively, and PLBF has 1.5 times more P_C/O_ than PLLA.

The decrease in CDP and OP of PLGA films is because the PGA chain segment possesses high gas barrier properties [45]. It should be noted, however, that in PLBF, the PBF chain segment has similar gas barrier properties to PBS [46]. Furthermore, the PBF chain segment contains unsaturated carbon–carbon double bonds and a stable trans double bond structure, which makes PLBF less susceptible to cis-trans isomerization, resulting in PLBF being more rigid and less susceptible to gas passage, thereby promoting a significant improvement in the barrier properties of PLBF. The size of gas molecules can also affect their solubility and diffusion in the polymer; O_2_ molecules have a diameter of 0.346 nm [47], while CO_2_ molecules have a diameter of 0.280 nm [48]. The large size of O_2_ molecules makes it more difficult to pass through PLBF copolymer films, and thus, P_C/O_ is enhanced.

### 3.2. Characteristics of Mushrooms

#### 3.2.1. Headspace Analysis

**Figure 3:**
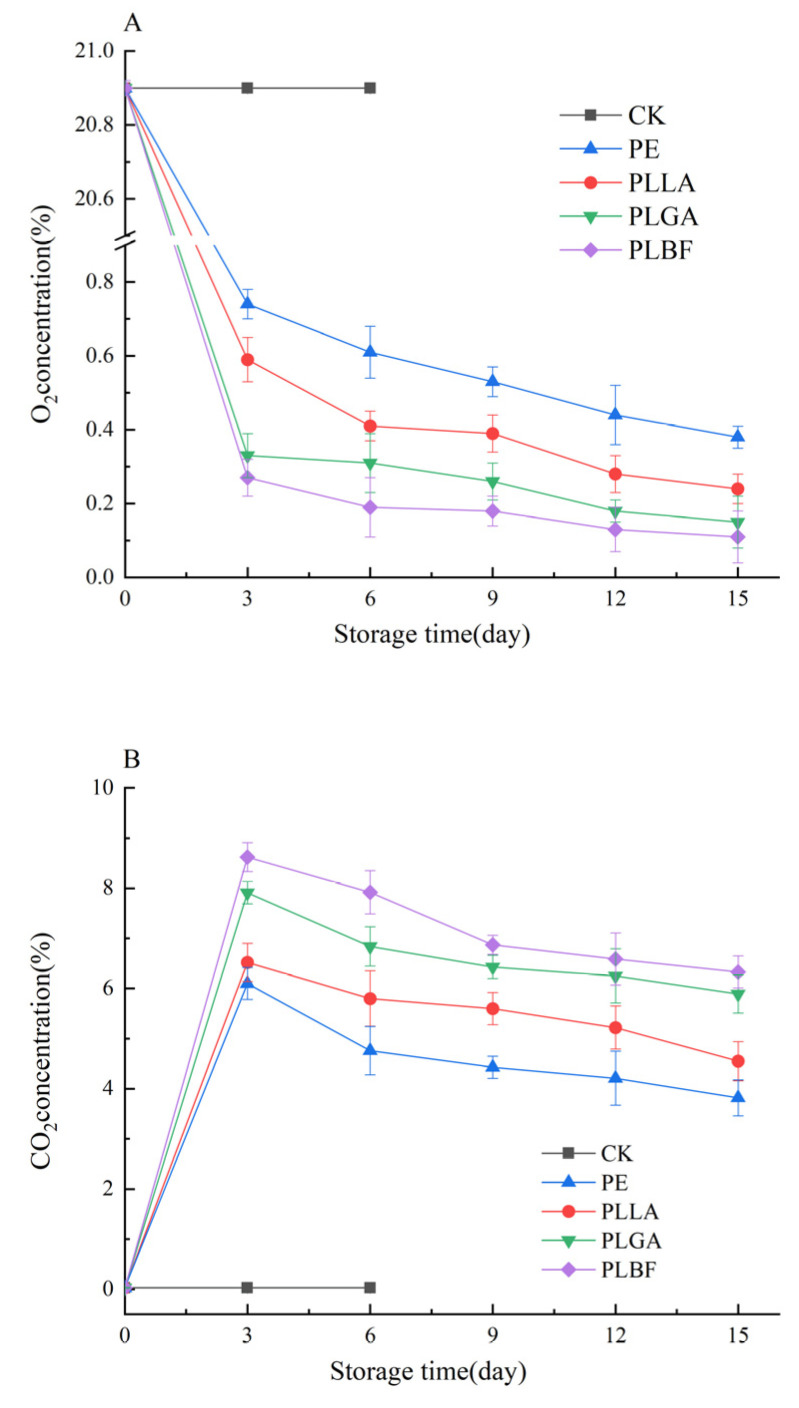
Changes in gas components O_2_ (**A**) and CO_2_ (**B**) of each packaging group during storage.

Figure 3A shows the variation pattern of O_2_ inside each package group with storage time. The O_2_ concentration of all four package treatments, PE, PLLA, PLGA, and PLBF, showed a sharp decrease in O_2_ concentration during 3 days of storage. The oxygen concentration fluctuated between 0.74%–0.38%, 0.59–0.24%, 0.74–0.38%, 0.33–0.15%, and 0.27–0.11%, respectively. Typically, lower oxygen concentrations slow the respiration rate of mushrooms, reducing changes in physiological structure and extending shelf life. In contrast, the respiration of mushrooms was first inhibited in the PLBF package group due to its lowest OP. Due to the relatively close O_2_ concentrations in each sample package, the effects on the weight loss of mushrooms may not differ greatly after reaching equilibrium. A similar study noted that the O_2_ concentration in the package was less than 1% on the 15th day but still better maintained the quality of edible mushrooms better and prolonged storage to 25 days [49].

As shown in Figure 3B, the CO_2_ concentration inside the packaging with PLBF film was higher than that inside the packaging with the other films. This was attributed to the lower CDP of PLLA films incorporated with PBF. In general, a CO_2_ concentration lower than 12% could reduce the growth and reproduction of thermophilic bacteria, browning, and respiration rate of mushrooms [7] and help maintain the hardness of mushrooms. Moreover, a high CO_2_ concentration of over 2.5% was also beneficial in reducing the opening rate of mushrooms [50]. As the storage time increased (6 days-15 days), the PLBF treatment group still had the highest CO_2_ content, fluctuating between 6.33% and 7.92%, and the CO_2_ concentration in PLBF was significantly higher than that in the other three treatment groups throughout the storage period. The reasons for this were mainly due to the variability in the CO_2_ transmission properties of the different films.

#### 3.2.2. Weight Loss 

**Figure 4:**
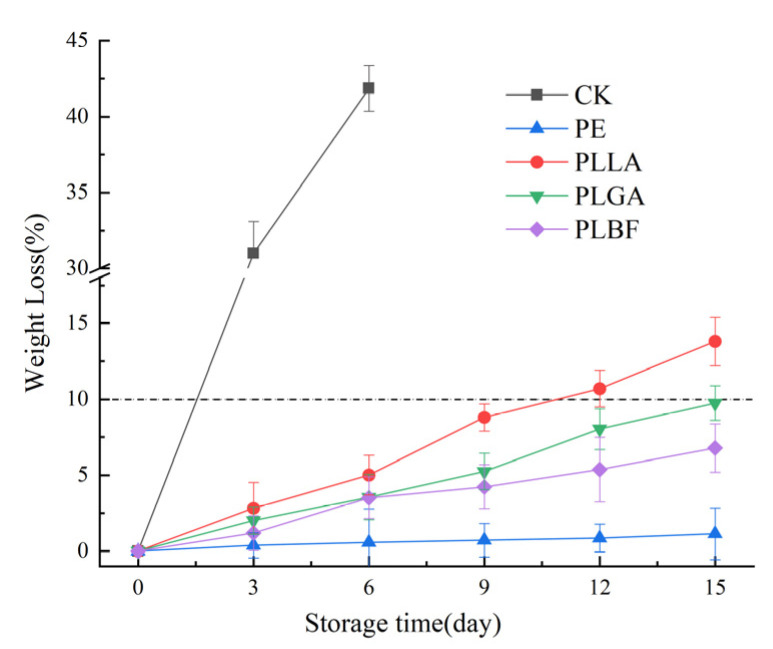
Changes in the weight of *Agaricus bisporus* in each packing group during storage.

The weight loss rate during storage can be utilized as an essential indicator to determine white mushroom freshness due to their delicate tissue and high moisture content. Respiration and transpiration processes result in water loss and nutrient depletion, which causes white mushrooms to deteriorate during storage [51,52]. The weight loss rate of white mushrooms packaged in different films is presented in Figure 4. In all samples, weight loss increased as the storage period increased. The CK group could not control the dehydration and transpiration of the mushroom skin due to a lack of film protection, resulting in a mass loss rate of 30.99% on the third day of preservation, which was significantly higher than that of the other packaged groups.

The quality loss of mushrooms packaged in PE film was always at a low level, only 1.7% after 15 days of storage, which was significantly lower than that of the PLLA (13.8%), PLGA (9.75%), and PLBF groups (6.78%), which was mainly due to the low water vapor permeability of the PE film, which resulted in a high humidity environment inside the package and reduced transpiration [28,53]. However, after 3 days of storage of *Agaricus bisporus*, water droplets could be found on both the film and *Agaricus bisporus* in the PE group packaging, and with the extension of the storage period, a large number of water droplets condensed to produce condensation, and condensation inside the packaging would promote the growth of microorganisms and accelerate the spoilage of *Agaricus bisporus*.

On the 15th day of storage, no condensation occurred inside the PLGA and PLBF groups, and the weight loss rate of the PLBF group was 6.78%, which was significantly lower than that of the PLGA group (9.75%), indicating that the change in weight loss rate was closely related to the packaging material. In addition to these factors, the change in the weight loss rate of mushrooms during storage was also related to other factors, such as the release of CO_2_ due to respiration, transpiration, and the role of microorganisms [50,54]. To a certain extent, packaging with a modified atmosphere could reduce water loss from produce.

#### 3.2.3. Browning Index

**Figure 5:**
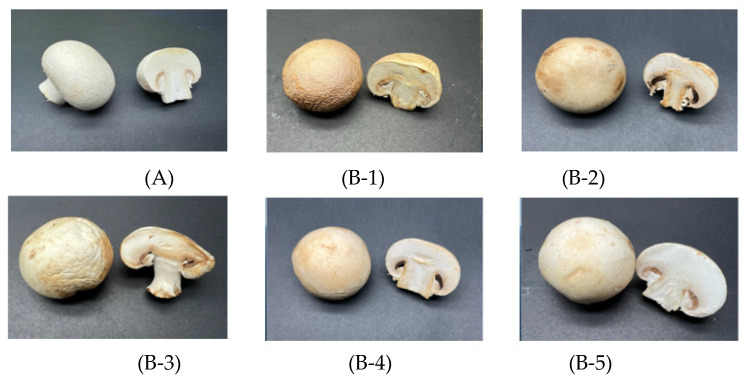
Surface and section images of *Agaricus bisporus* of CK (**A**) in 0 days and CK (**B-1**), PE (**B-2**), PLLA (**B-3**), PLGA (**B-4**), and PLBF (**B-5**) at 15th days.

**Figure 6:**
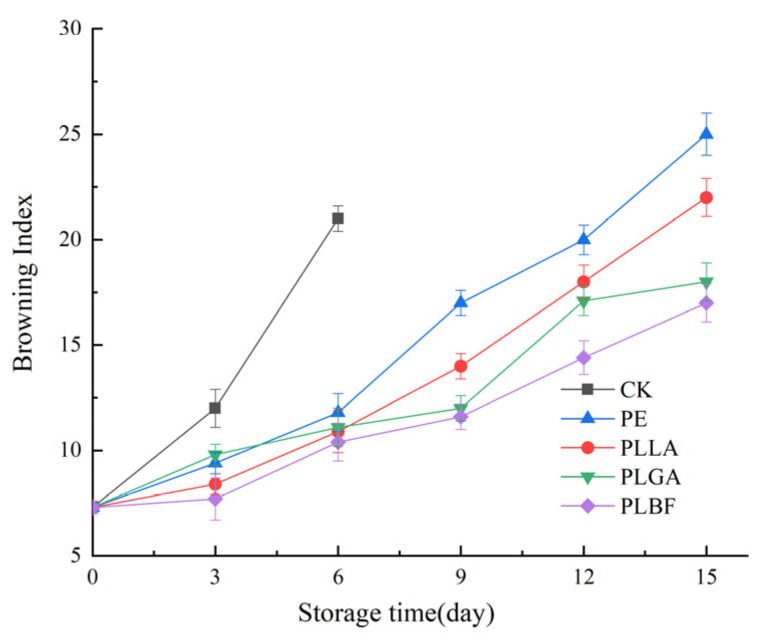
Changes of BI value of *Agaricus bisporus* in each packing group during storage.

*Agaricus bisporus* is prone to browning after harvest, and the degree of browning increases as the time after harvest increases (Figure 5). It is generally believed that the browning of fresh *Agaricus bisporus* is mainly caused by enzymatic browning, and during storage, white mushrooms turn from white to brown or black due to enzymatic browning of the substrates, which negatively affects their commercial value [55]. Figure 6 illustrates the impact of packaging on BI when mushrooms are packaged in different films. All mushrooms showed an increase in BI during storage, indicating that their whiteness was reduced. Figure 6 shows that the BI of both the CK group and the packaged group increased as the storage period increased. On day 3 of storage, there was a significant difference in BI between the CK group and the packaged group, with the control group having a significantly higher BI than the packaged group. After day 9 of storage, the BI of the PLBF group was significantly lower than that of the PE, PLLA, and PLGA groups, and this advantage was maintained until the end of the storage period. The high BI value in the PE group is probably a result of high relative humidity in the package, which resulted in high bacterial growth, reproduction, and number, and therefore *Agaricus bisporus* substrates were degraded and the oxidation of phenolic substances by tyrosinase produced brown substances [56].

#### 3.2.4. Total Phenolics and Ascorbic Acid

**Figure 7:**
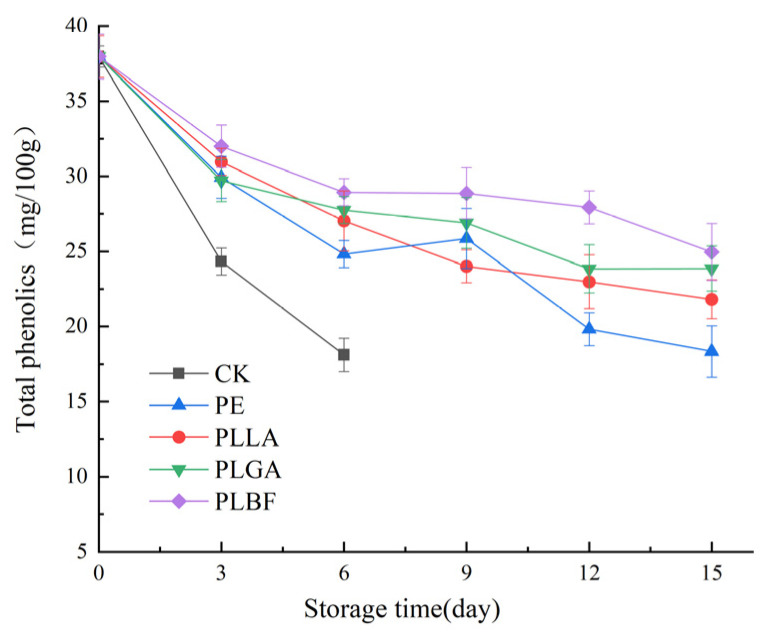
Changes of total phenolic content of *Agaricus bisporus* in each packaging group during storage.

Phenolic compounds are major antioxidant components in mushrooms and other plants. They are important secondary metabolites that scavenge free radicals and chelate metals and reduce lipoxygenase activity, which is essential in resisting adverse external environmental stresses, internal oxidative stresses, and pathogenic bacterial growth infestation. Oxidation of phenolics is the main factor in the postharvest browning of *Agaricus bisporus*, which adversely affects its commercial value [57,58]. As shown in Figure 7, mushrooms’ total phenolic (TP) content was reduced differently in different packages. The TP content in the control group was the lowest at the end because polyphenols might convert to quinones by polyphenol oxidases and react with oxygen to form brown-colored compounds [59].

After 6 days, the TP content was reduced by 52.34%, 34.64%, and 28.80% in the CK, PE, and PLLA groups, respectively, compared to the initial TP content, while the PLGA and PLBF groups had significantly higher TP contents than the other groups. Throughout the storage period, the decreasing trend of phenolics in the samples of the PLBF group was smoother and consistently higher than the other packaging groups. This indicates that the low O_2_ and high CO_2_ gas environment formed inside the PLBF package can effectively inhibit the activity of polyphenol oxidase, thus reducing the oxidation of polyphenols and inhibiting the decrease in TP content during storage of *Agaricus bisporus*.

**Figure 8:**
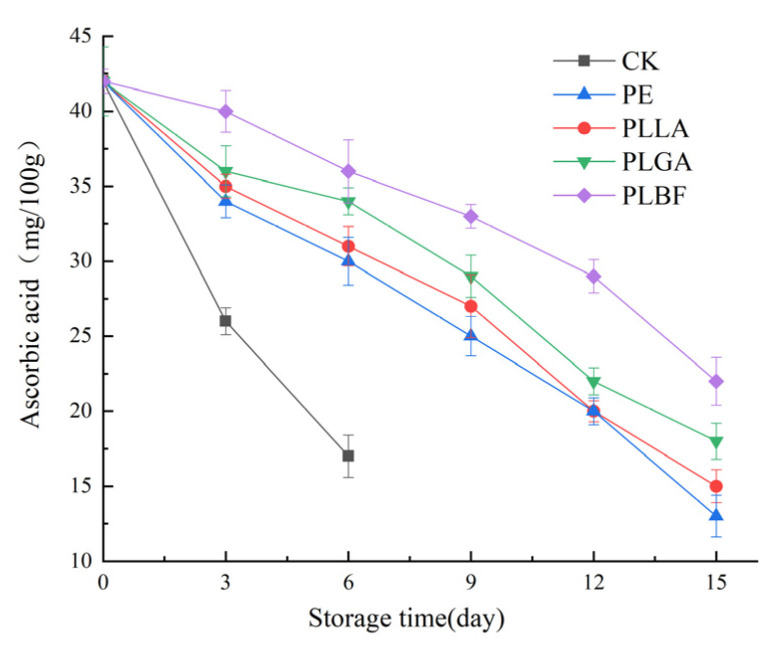
Changes of total ascorbic acid of *Agaricus bisporus* in each packaging group during storage.

Ascorbic acid (AA) is a powerful endogenous antioxidant, that plays a vital role in removing reactive oxygen species from fruits and vegetables, and is related to the senescence of mushrooms. As shown in Figure 8, the AA content of all treatment groups showed a decreasing trend during storage, among which the AA content of the CK group decreased rapidly and was significantly lower than that of the other four treatment groups throughout storage. The AA content in the PE, PLLA, and PLGA treated groups decreased rapidly to 13 mg/100 g, 15 mg/100 g, and 18 mg/100 g, exhibiting a decrease of 69.04%, 62.2%, and 57.1% at the end of storage, respectively. The AA content of the PLBF group decreased relatively slowly with the extension of storage time, and the AA content of this group was higher than that of the other three packaged groups throughout storage and remained at 22.01 mg/100 g at the end of storage. The loss of AA was better inhibited in the PLBF treatment group, which may be related to the higher CO_2_ content in the package, and the higher concentration of CO_2_ in the bag could form hydrogen bonds between the cell wall and polar groups, preventing the expression of certain enzymes and AA reduction [19]. Furthermore, AA can reduce reactive oxygen species (ROS) through the ascorbate-glutathione cycle or react with reactive forms of oxygen such as ozone to inhibit tissue senescence and browning [60], and the rapid loss of AA in the three packaging groups PE, PLLA, and PLGA may be related to the rate of reactive oxygen species production in these three groups.

#### 3.2.5. Malondialdehyde Content and Electrolyte Leakage Rate

**Figure 9:**
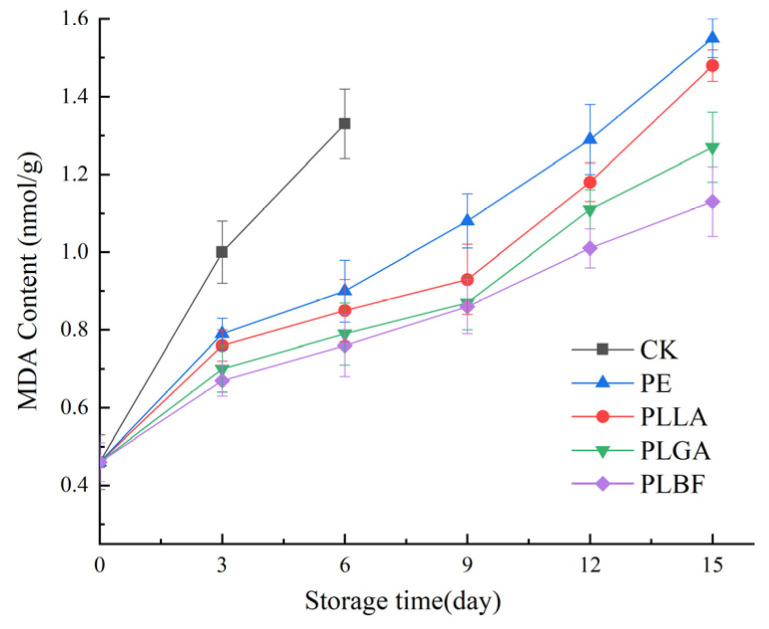
Changes of MDA of *Agaricus bisporus* in each packing group during storage.

**Figure 10:**
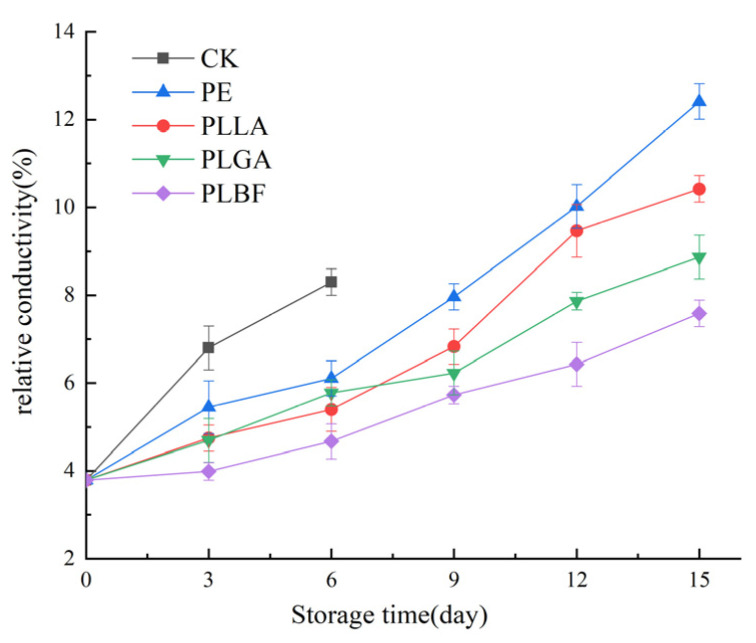
Changes of relative conductivity of cell membrane of *Agaricus bisporus* in each group during storage.

Electrolyte leakage(EC) and malondialdehyde (MDA) content indicate the extent of damage and membrane lipid peroxidation in *Agaricus bisporus* cell membranes [61,62], which is indicative of the quality of *Agaricus bisporus* as is evident from Figure 9 and Figure 10, which show that the electrolyte leakage and MDA of all samples raised gradually during storage. The explanation for this is that the quality of *Agaricus bisporus* continues to decline with storage time, with soft wilting and browning, leading to oxidation of the lipids and efflux of cytoplasm from *Agaricus bisporus*.

On the 6th day of storage, the relative conductivity and MDA content of the CK group increased to 8.3% and 1.33 nmol/g, respectively. The relative conductivity of PE and PLLA increased to 12.4% and 10.4% on the 15th day of storage, while the MDA content increased to 1.55 nmol/g and 1.48 nmol/g, respectively. Because an outer packaging does not safeguard CK, while the PE and PLLA groups have a high O_2_ content inside the bags, the metabolically active cells of *Agaricus bisporus* are under stress from the gaseous environment, which leads to lipid oxidation within the cell, further disrupting the integrity of the cell and leading to an increase in relative conductivity. In addition, the hyperoxic environment also accelerates the oxidation of TP and AA, leading to a sharp rise in MDA.

On the 15th day of storage, the relative conductivity of PLGA and PLBF was 8.8% and 7.6%, and the MDA was 1.27 nmol/g and 1.13 nmol/g, respectively, markedly inferior to the other groups. This can be attributed to the fact that the low O_2_ and higher CO_2_ environment created by the PLGA and PLBF groups effectively inhibited the respiratory metabolism of *Agaricus bisporus*. It is worth mentioning that PLBF maintained the lowest relative conductivity and MDA values compared to the other groups, which indicates that the storage atmosphere created by PLBF delays the oxidation of *Agaricus bisporus* membrane lipids while effectively maintaining the integrity of *Agaricus bisporus* cells.

#### 3.2.6. Enzyme Activity

The activities of catalase (CAT) and superoxide dismutase (SOD) enzymes are intimately related to the degree of damage of the organism, which can directly affect the quality of *Agaricus bisporus* [11]. Antioxidant enzymes such as CAT and SOD play a crucial role in antioxidant defense during the storage of fruit and vegetables [9]. 

**Figure 11:**
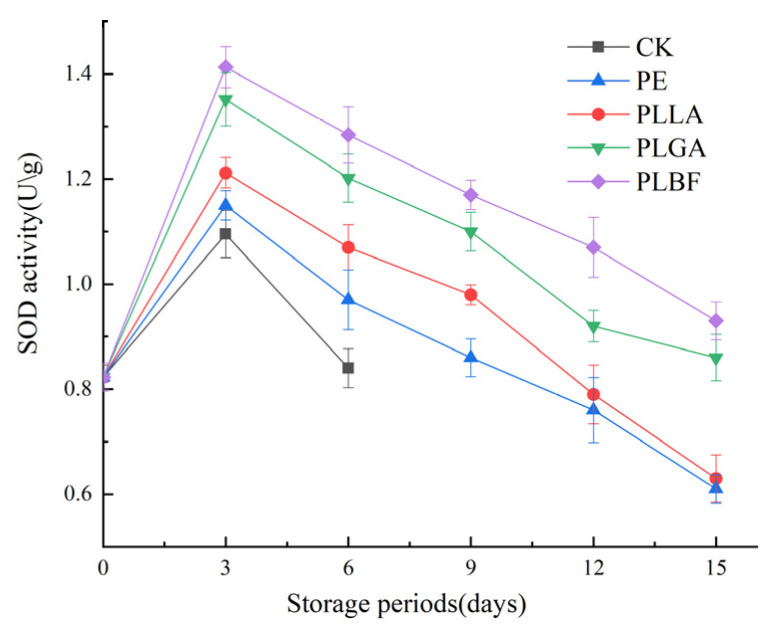
Changes of SOD activity of *Agaricus bisporus* in each packing group during storage.

**Figure 12:**
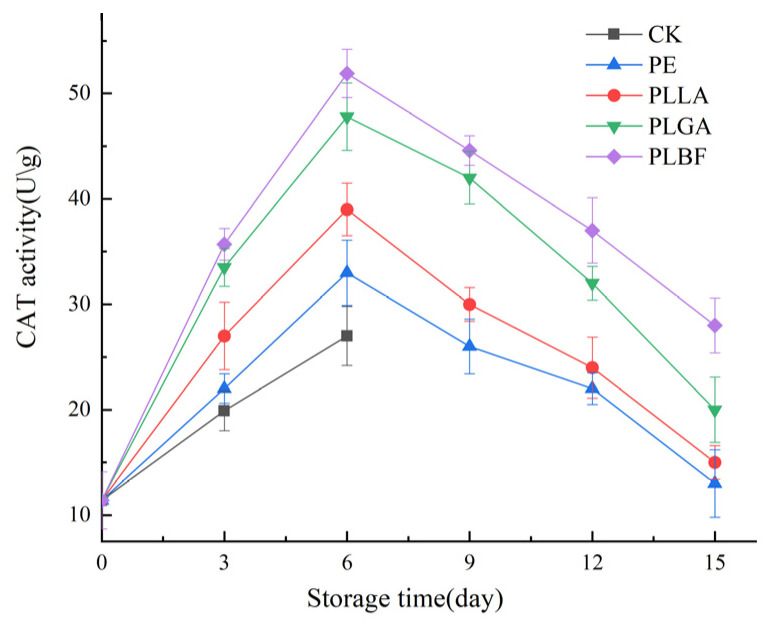
Changes of CAT activity of *Agaricus bisporus* in each packing group during storage.

The changes in CAT and SOD enzyme activities are summarized in Figure 11 and Figure 12. CAT and SOD enzyme activities showed an overall increasing trend at the beginning of storage, and the enzyme activity decreased as the storage time increased. This can be explained by the inability of the mycelium of *Agaricus bisporus* to be supplied with water and inorganic and organic nutrients after harvest. They resulted in the badlands’ stress of the SOD and CAT enzymes, which may have contributed to the increase in CAT and SOD enzyme activities [63]. In the later stages of storage, the quality of *Agaricus bisporus* dropped, and the activity of CAT and SOD enzymes continued to decline.

The CAT and SOD activities of PLBF were higher during storage. On the 15th day of storage, the CAT activities of the PE, PLLA, and PLGA groups were only 13 U/g, 15 U/g, and 20 U/g, and the SOD activities were only 0.61 U/g, 0.64 U/g, and 0.86 U/g, respectively, which were substantially weaker than those of the PLBF group. The CAT and SOD activities of the PLBF group were as high as 28 U/g and 0.93 U/g, respectively. These manifestations indicate that the gas fraction formed by PLBF (0.11% O_2_ + 6.3% CO_2_) can effectively maintain the activity of CAT and SOD, slow the oxidation and ageing process of *Agaricus bisporus*, and maintain the excellent quality of *Agaricus bisporus*.

#### 3.2.7. Relevance Analysis

Correlation analysis between the gas composition in the package and the quality of *Agaricus bisporus* was performed. As shown in Table 4, there was a close relationship between the gas composition in the package and the storage quality of *Agaricus bisporus*. In the results obtained, the O_2_ concentration in the bag was highly significantly correlated with the TP of *Agaricus bisporus* (*p <* 0.01) and with the BI and EC of *Agaricus bisporus* in the bag (*p <* 0.05). The CO_2_ concentration in the bag was highly significantly correlated (*p <* 0.01) with BI and significantly correlated with TP, AA, MDA, EC, and SOD of *Agaricus bisporus*. In addition, O_2_ inside the package had a strong correlation of −0.924, 0.930, −0.875, and −0.901 with AA, MDA, CAT, and SOD of *Agaricus bisporus*, respectively, and CO_2_ inside the package had a strong correlation of 0.931 with CAT of *Agaricus bisporus*. These results further suggest that regulating gas composition inside the pouch by changing the OTR and CDTR of PLLA-based films can further regulate the changes in postharvest physiological and biochemical indices, sensory traits, and antioxidant enzymes of *Agaricus bisporus* and improve its postharvest quality and shelf life of. This is because the TP and AA of *Agaricus bisporus* are easily oxidized [64], and higher O_2_ concentrations in the storage environment of *Agaricus bisporus* lead to higher BI, EC, and MDA and lower CAT and SOD [65,66]. Higher CO_2_ concentrations and lower O_2_ concentrations are more favorable for maintaining the quality of *Agaricus bisporus* [11].

## 4. Conclusions

PLLA film cooperative polymerization with OBF or GA showed a positive preservation effect on the cold storage of *Agaricus bisporus*. Compared with the control, PE, and PLLA film packaged groups, copolymer-modified PLLA films could not only decrease the rate of weight loss, browning index, and electrolyte leakage rate of *Agaricus bisporus* but also decrease the contents of MDA. Meanwhile, TP and AA increased in the PLGA and PLBF groups. Additionally, PLLA films incorporated with OBF increased the activity of SOD and CAT in mushrooms during storage. The results showed that, in two copolymer-modified PLLA films, PLBF packaging had lower OP and CDP. It effectively reduced the weight loss rate to less than 7% at the end of storage. In addition, it created the optimal gas composition for *Agaricus bisporus* preservation, 0.27–0.11% O_2_ and 6.33~7.92% CO_2_, which significantly reduced the respiration metabolism, membrane lipid peroxidation, and postharvest senescence of *Agaricus bisporus* (*p <* 0.05). It maintained the highest level of market acceptability (*p <* 0.05). The shelf life of *Agaricus bisporus* in PLBF reached 15 days. This study provides a feasible solution to reduce postharvest senescence and maintain the quality of *Agaricus bisporus*. 

## Figures and Tables

**Table 1:** Mechanical properties of PLLA/PLGA/PLBF modified atmosphere breathing membrane.

Sample Name	*x*/LA wt/wt	*x*/LA *^a^* wt/wt	Mn *^b^*	Pd *^b^*
PLLA	—	—	40,375	2.33
PLGA	8/92	7.5/92.5	45,296	2.02
PLBF	8/92	9/91	58,359	1.97

*x* means GA unit or BF unit. *^a^* Determined by ^1^H NMR based on GA unit (2H, 4.85 and 4.60 ppm) and lactic acid (LA) unit (1H, 5.15 ppm) of the PLGA copolymers. Determined by ^1^H NMR based on BF unit (1H, 6.88 and 4.23ppm) and lactic acid (LA) unit (1H, 5.15 ppm) of the PLBF copolymers. *^b^* Determined by GPC.—means this is a pure PLLA with no other components present.

**Table 2:** Mechanical properties of PLLA/PLGA/PLBF modified atmosphere breathing membrane.

Films	*E*/(MPa)	σ_t_/(MPa)	ε_b_/(%)
PLLA	1352 ± 16.7	40.3 ± 2.0	5.7 ± 1.1
PLGA	525 ± 8.6	23.3 ± 3.1	121.1 ± 10.4
PLBF	559 ± 6.4	25.6 ± 2.4	139 ± 15.8

**Table 3:** Gas barrier properties of PLLA/PLGA/PLBF modified atmosphere breathing membrane.

Films	CDP (10^−12^·cm^3^/m·s·Pa)	OP (10^−12^·cm^3^/m·s·Pa)	P_C/O_
PLLA	4.51 ± 0.35 ^A^	1.50 ± 0.23 ^A^	3.0
PLGA	2.55 ± 0.23 ^B^	0.69 ± 0.13 ^B^	3.7
PLBF	2.08 ± 0.12 ^B^	0.46 ± 0.12 ^B^	4.5

Note: Different capital letters in the same column with shoulder markers indicate significant difference (*p <* 0.05), same capital letters in the same column indicate non-significant difference (*p* > 0.05).

**Table 4:** Correlation analysis between gas composition in packing and postharvest quality of *Agaricus bisporus*.

	O_2_	CO_2_	Wl	BI	TP	AA	MDA	EC	CAT	SOD
O_2_	1	−0.972 *	−0.507	0.987 *	−10.00 **	−0.924	0.930	0.989 *	−0.875	−0.901
CO_2_		1	0.296	−0.996 **	0.969 *	0.962 *	−0.984 *	−0.984 *	0.931	0.978 *
Wl			1	−0.377	0.511	0.184	−0.158	−0.398	0.082	0.094
BI				1	−0.984 *	−0.944	0.966 *	0.987 *	−0.904	−0.957 *
TP					1	0.926	−0.929	−0.990 **	0.878	0.897
AA						1	−0.989 *	−0.970 *	0.993 **	0.954 *
MDA							1	0.968 *	−0.978 *	−0.987 *
EC								1	−0.936	−0.935
CAT									1	0.942
SOD										1

Note: ** represents extremely significant correlation (*p* < 0.01); * represents significant correlation (*p* < 0.05).

## Data Availability

Data is contained within the article.
